# Probing the Behaviors of Gold Nanorods in Metastatic Breast Cancer Cells Based on UV-vis-NIR Absorption Spectroscopy

**DOI:** 10.1371/journal.pone.0031957

**Published:** 2012-02-22

**Authors:** Weiqi Zhang, Yinglu Ji, Jie Meng, Xiaochun Wu, Haiyan Xu

**Affiliations:** 1 Institute of Basic Medical Sciences, Chinese Academy of Medical Sciences and Peking Union Medical College, Beijing, People's Republic of China; 2 CAS Key Laboratory of Standardization and Measurement for Nanotechnology, National Center for Nanoscience and Technology, Beijing, People's Republic of China; University of Helsinki, Finland

## Abstract

In this work, behaviors of positively-charged AuNRs in a highly metastatic tumor cell line MDA-MB-231 are examined based on UV-vis-NIR absorption spectroscopy in combination with inductively coupled plasma mass spectrometry (ICP-MS), transmission electron microscopy (TEM) and dark-field microscopic observation. It is found that characteristic surface plasmon resonance (SPR) peaks of AuNRs can be detected using spectroscopic method within living cells that have taken up AuNRs. The peak area of transverse SPR band is shown to be proportionally related to the amount of AuNRs in the cells determined with ICP-MS, which suggests a facile and real time quantification method for AuNRs in living cells. The shape of longitudinal SPR band in UV-vis-NIR spectrum reflects the aggregation state of AuNRs in the cells during the incubation period, which is proved by TEM and microscopic observations. Experimental results reveal that AuNRs are internalized by the cells rapidly; the accumulation, distribution and aggregation of AuNRs in the cells compartments are time and dose dependent. The established spectroscopic analysis method can not only monitor the behaviors of AuNRs in living cells but may also be helpful in choosing the optimum laser stimulation wavelength for anti-tumor thermotherapy.

## Introduction

Gold nanorods (AuNRs) have been widely explored in biosensing, imaging, drug delivery and photothermal therapy because of their special physicochemical properties [1]–[6]. The applications of AuNRs in biomedicine were firstly based on AuNRs themselves in bulk were inert and nontoxic towards mammalian cells [7]–[8]. Current researches with AuNRs in nanomedicine are mostly focused on employing AuNRs as delivery vehicles, imaging agents and thermo-therapeutic modules [2], [5], [8]–[10]. Increasing experimental results indicated that AuNRs had attractive and promising application potentials in RNAi therapy, drug delivery across blood brain barrier, cancer diagnosis and imaging, and laser stimulated thermotherapy against cancers [1], [11]–[13]. However, the behaviors of AuNRs in cells as well as influences that the AuNRs exerted on cells function such as cells viability and cell cycles were far from adequate and required further elucidation urgently [8].

The behaviors of AuNRs encountering with cells in vitro could be basically divided into two aspects: outside the cells (in the cell culture system) and inside the cells. There were a variety of ions, saccharides, amino acids as well as proteins in serum containing media (SCM). It has been generally accepted that nanoparticles would be coated by a corona of serum proteins when they were introduced into the SCM [14]–[15]. This protein corona could further dictate the fate of the nanoparticles in cells including uptake and cellular location [15]. Recently, Albanese *et al* found that single and aggregated gold nanoparticles had different uptake patterns while the aggregation did not cause significant cytotoxicity [16]. The phenomenon suggested that the aggregation state of nanoparticles in culture media should be handled cautiously when evaluating the behaviors of nanoparticles in mammalian cells in vitro.

Following the uptake of the AuNRs, their interactions with cellular membrane, compartments and molecules have attracted growing attentions [9], [17]–[18]. A clear understanding of the interactions was still lacking but undoubtedly would be beneficial in designing AuNRs as drug or gene delivery vectors, thermo-therapeutic modules as well as alleviating their side effects such as cytotoxicity [5], [18]–[19]. During the past few years, large efforts have been made to probe the uptake and location of AuNRs in mammalian cells mainly based on various imaging techniques and inductively coupled plasma mass spectrometry (ICP-MS) [15], [20]–[22]. For example, using transmission electron microscopy (TEM), one could visualize the uptake and location of AuNRs in cells at the single nanoparticle level. In combination with ICP-MS, Au content in cells could be detected with high sensitivity [23]–[24]. Several studies have shown that AuNRs were endocytosed and trapped in vesicles or lysosomes in mammalian cells [5], [10], [15], [19], [25]. When conjugated with nuclear-targeting peptides, AuNRs were observed located in the nucleus [26]. The different location of AuNRs may have its specific application potentials. When AuNRs were used as siRNA or gene delivery vectors, the cytoplasmic location would facilitate the gene silencing as the gene silencing machinery was mainly in cytoplasm, while the nuclear location would benefit the exogenous gene expression as the gene expression was mainly regulated in nucleus [3], [26].

However, sample preparation for TEM was time consuming and a very limitation was that only a limited amount of cells were involved in the TEM observation. In ICP-MS analysis cells had to be destructed and only gold in ionic form could be detected [23]. Consequently, establishing a quantification system using existing technologies to simultaneously determine the amount and aggregation state of AuNRs in living cells was of great significance.

AuNRs had two distinctive absorption bands in their absorption spectra; the one around 520 nm was called transverse surface plasmon resonance (TSPR) band and the other one which could be tuned according to the aspect ratio in the near infrared spectral region was referred to as longitudinal surface plasmon resonance (LSPR) band. The LSPR band was highly sensitive towards dielectric change of surrounding environment and distance and orientation between nanorods. Adsorption of proteins on AuNRs induced a red shift of the LSPR peak, and aggregation of AuNRs resulted in broadening and a shift of LSPR band [1], [25]. Recently, Xia's group reported a simple spectroscopic method to indirectly quantify the uptake of AuNRs by cells (breast cancer cell line, SK-BR-3) based on LSPR peak absorbance variation in culture media before and after the cell culture [27]. Additionally, due to distinct peak positions of gold nanospheres and AuNRs, their uptake amount could be differentiated when cells were incubated with a mixture of these gold nanostructures. This simple method provided an easy way to quantify the amount of Au nanostructures up taken by cells. However, we noticed two drawbacks in this method. One is that the aggregation status of the AuNRs in the living cell is lacking; and the other is the effect of aggregation on detection accuracy as both the intensity and peak of the LSPR band change with aggregating. In present work, we address these two issues by extending this strategy to living cells. The absorption area of TSPR bands in cells are used to directly quantify the amount of the AuNRs internalized by cells. The results are further corroborated by ICP-MS, TEM and dark field microscopy. In addition, the shape of LSPR band of AuNRs in cells is used to trace the aggregation status of the AuNRs in living cells.

## Results and Discussion

### Characterization of AuNRs in Serum Culture Medium

**Figure 1:**
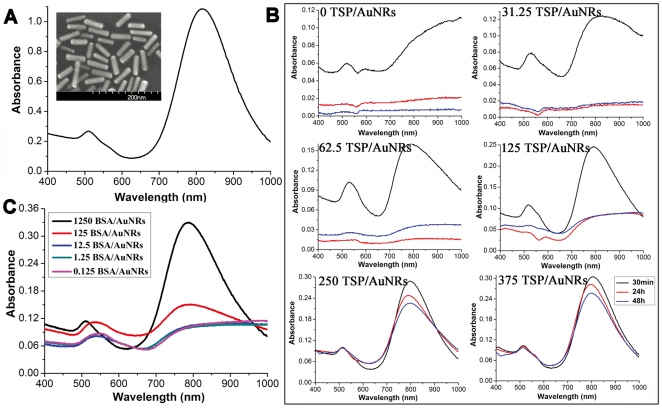
Characterization of AuNRs dispersed in water and culture media. (A) The UV-vis-NIR absorption spectrum of AuNRs dispersed in water. The inserted SEM image shows the morphology of AuNRs deposited from the aqueous solution. (B) Absorption spectra of AuNRs dispersed in serum containing media (SCM) with 0%, 2.5%, 5%, 10%, 20% and 30% of fetal bovine serum (FBS) as a function of incubation time. Concentration of AuNRs in the media was 120 pM. The corresponding ratios of total serum proteins (TSP) to AuNRs (TSP/AuNRs) were 0, 31.25, 62.5, 125, 250, and 375. (C) The absorption spectra of AuNRs dispersed in basic media containing different content of only bovine serum albumin (BSA) for 30 minutes. The ratios of BSA to AuNRs (BSA/AuNRs) were 1250, 125, 12.5, 1.25 and 0.125.

The original CTAB (cetyltrimethylammonium bromide)-coated AuNRs were synthesized using well-developed seed-mediated growth. They were further sequentially coated by poly (sodium-p-styrenesulfate) (PSS) and poly (diallyldimethyl ammoniumchloride) (PDDAC) molecules through electrostatic layer-by-layer assembly technique as described in our previous work [28]. This modification strategy increased the stability of AuNRs in culture media meanwhile reduced their cytotoxicity caused by CTAB molecules desorbed from the AuNRs surface [4], [29]. The PDDAC-coated AuNRs were characterized by scanning electron microscopy (SEM) and UV-vis-NIR absorption spectrophotometer (shown in **Figure 1A**). The aspect ratio (length vs. width) was estimated to be 4.3; and the TSPR and LSPR peaks were at 512 nm and 817 nm, respectively. **Figure 1B** presents the effects of serum on absorption spectra of AuNRs dispersed in SCM. The ratios of total serum proteins (TSP) to AuNRs (TSP/AuNRs) were used to study the influences of serum content on stability of AuNRs in the SCM. In the basic medium which was serum free, AuNRs aggregated quickly with a severe broadening of the LSPR band; after 30 minutes incubation, the majority of the AuNRs precipitated from the medium due to too large size of the aggregates as evidenced by the great loss of AuNRs absorbance. The culture media were full of various ions which could screen the electrostatic repulsion between AuNRs and finally resulted in the aggregation of them in the basic media [4]. When serum was supplemented in the culture media, the stability of AuNRs in the SCM was improved as a proportional function of serum concentration. The AuNRs were well dispersed in the SCM when the TSP/AuNRs ratio was > 125. Adsorption of serum proteins in SCM to AuNRs surface has been suggested to stabilize the AuNRs [4], [29]. When the proteins were not enough to coat each individual nanorod, AuNRs had to share the same proteins which bridged the AuNRs together [30]. Hence adequate serums were required to obtain a stable AuNRs dispersion in SCM. To further confirm this, the UV-vis-NIR spectra of AuNRs dispersed in an only bovine serum albumin (BSA) containing culture medium were measured. A similar phenomenon was observed. When the ratio of BSA to AuNRs (BSA/AuNRs) in the solution was less than 125 BSA/AuNRs, most AuNRs existed in aggregated status as the LSPR band broadened significantly with great reduction in intensity. Further increasing the BSA/AuNRs ratio, the absorption features of individual AuNRs were recovered (**Figure 1C**). Notice that at same BSA/AuNRs ratio (125 BSA (or TSP)/AuNRs), the LSPR band in the only BSA containing culture media is much broadened than that in SCM, indicating a better dispersion of the AuNRs in the latter media. This was presumably the result of the relatively poor capability of only BSA in maintaining the AuNRs stability in culture media as a combination of serum proteins constructed the corona of nanoparticles exposed to the serum [31]–[32].

Dispersion status of AuNRs in SCM was also incubation time-dependent. As seen in **Figure 1B**, when the ratio of TSP/AuNRs in the media was below 125, the LSPR band was reduced greatly after 24 or 48 h incubation, indicating further aggregation. At TSP/AuNRs ratio ≥250, the LSPR band was better maintained in SCM. In the media, nanoparticles (especially large or heavy nanoparticles) experienced a sediment effect during the extended incubation due to the gravity [33]. The sedimentation would be markedly augmented when slight aggregation of AuNRs occurred in SCM with low TSP/AuNRs ratio. These findings remind us when assessing the interaction between AuNRs and cells with dosage variation, the aggregation state of AuNRs should be taken into consideration as the commonly used serum content in culture media was fixed (10% serum). By UV-vis-NIR absorption spectroscopy, AuNRs concentration and incubation time were carefully selected to avoid aggregation problems in the following cellular experiments.

### UV-vis-NIR spectroscopy and ICP-MS with AuNRs-incubated cells

**Figure 2:**
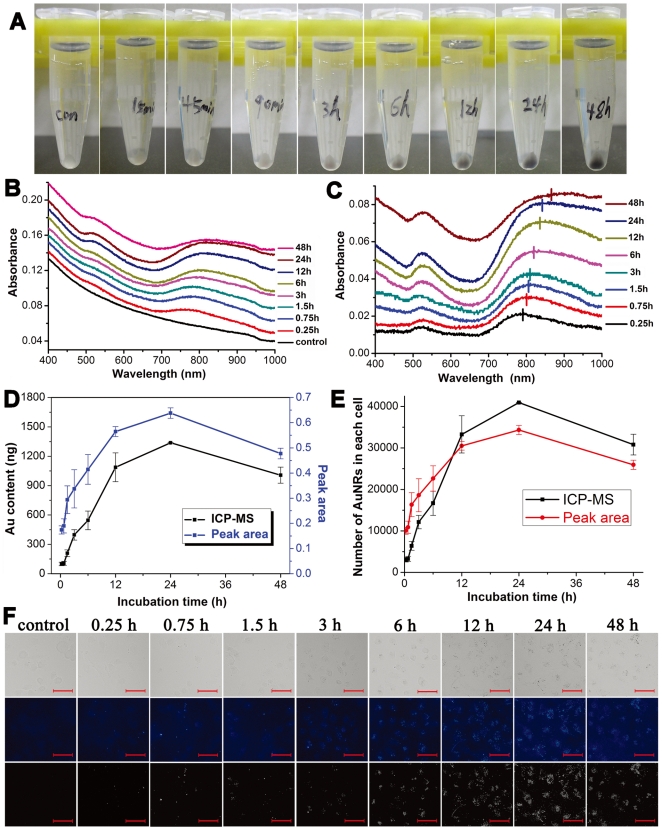
Analysis of AuNRs internalized by MDA-MB-231 cells vs incubation time. (A) Pellets of the cells incubated with AuNRs for 0.25, 0.75, 1.5, 3, 6, 12, 24, and 48 hours. Cells with no treatment are control. (B) The representative UV-vis-NIR absorption spectra of the cells incubated with AuNRs for different hours. The curves are offset for clarity. (C) The absorption spectra of AuNRs in cells. The absorption from the control cells is subtracted as background and the spectra are offset for clarity. (D) Comparison between results obtained from ICP-MS and TSPR peak area value. (E) The data acquired from the ICP-MS and TSPR peak area is converted to number of AuNRs contained in each cell. (F) Bright and dark field microscopic observation of the cells incubated with AuNRs for different hours (TSP/AuNRs is 250). The first line is the bright field microscopic observation. The second and third line is the dark field microscopic images with and without false color. The scale bar represents 50 µm.

MDA-MB-231 cells were incubated with 60 pM of AuNRs (250 of TSP/AuNRs) for different hours. At each designated time point, the cells were detached and centrifuged to obtain cell pellets. The pellets were dispersed in PBS and 1.5×10^5^ of the cells were subjected to spectroscopic characterization. Same number of the cells with no treatment was set as a control for the background subtraction. As shown in **Figure 2A**, at the beginning of incubation, the color of the cell pellets resembled that of AuNRs dispersed in water. The appearance and gradual increase of black color in the cell pellets was observed after 3 h of incubation, implying the increased amount of engulfed AuNRs and/or their increased aggregation inside the cells during the extended incubation.

On the basis that AuNRs with this aspect ratio have been shown efficiently internalized by mammalian cells reported in literatures [19], [34] as well as in current experiments, and most of the cells themselves had very weak absorption in the near-infrared window, we hypothesized that if high content of AuNRs were taken up by the cells, the AuNRs-contained cells would display the characteristic absorption of the AuNRs in the near-infrared region. To verify the above hypothesis, cells were subjected to UV-vis-NIR spectroscopic measurements as shown in **Figure 2B**. The control cells were shown to have a weak absorbance in the near-infrared region which was a transparent window used for NIR imaging or thermotherapy [2], [5]. The AuNRs-incubated cells gained two absorption bands belong to AuNRs. As incubation time was extended, the two bands grew more legible. After subtracting the background absorbance from the control cells, spectra of AuNRs in the cells after different exposure times were presented in **Figure 2C**. The absorption spectra were still similar to AuNRs dispersed in water. However, the LSPR band exhibited a gradual red shift and unsymmetrical broadening to longer wavelength region within the incubation period. The red shift extent was proportionally associated with the color darkening of the cell pellets with incubation time increased. Hence the red shift may act as an indicator of AuNRs aggregation in the cells.

Because the shape of TSPR peak of AuNRs in cells was steadily maintained and the peak area exhibited an increasing trend within the incubation time, the area of TSPR band was calculated by a line segment connecting the start and end inflection points of the TSPR peak in the absorption spectrum (Figure S1). Then the calculation results were compared with analysis results of gold content in cells obtained from ICP-MS (**Figure 2D–E**). As seen in **Figure 2D**, the value of TSPR peak area exhibited the same tendency as a function of incubation time towards gold content in cells determined by ICP-MS. Results from both peak area calculation and ICP-MS showed the amount of AuNRs inside MDA-MB-231 cells was proportional to incubation time within 24 h; however, a slight decrease after 48 h incubation was observed. This reduction was presumably resulted from the AuNRs dilution by cells duplication, as doubling time for MDA-MB-231 cells was approximately 24 hours [35]–[36]. The exocytosis of AuNRs may also be attributed to the decrease [21] but not the main reason here as the concentration of the AuNRs in SCM after 48 h incubation was further decreased (**Figure S4**).

In order to further compare the two quantification methods, the data from TSPR peak area and ICP-MS were converted to number of AuNRs per cell using a calibration line shown in **Figure S2** and the calculation method reported in a reference [21], respectively (see Materials and Methods). The peak area was found to have an excellent linear relationship with the AuNRs concentration as shown in **Figure S2**. **Figure 2E** shows that at each incubation time point, AuNRs number per cell calculated from the calibration has a similar tendency to that calculated from ICP-MS results, though the result from spectroscopy-based method is 0.84–3.3 times higher than that from the ICP-MS. Interestingly, results from the spectroscopic method proposed by Xia *et al* to quantify AuNRs in the cells were 1.1–3.5 times higher than that from the ICP-MS [27], which was quite close to the outcome from our spectroscopic method. The difference between spectroscopy-based quantification and ICP-MS may arise from different detection sensitivities, because ICP-MS could detect gold down to pictogram scale. However, in comparison with ICP-MS, spectroscopic quantification of AuNRs was nondestructive; cells exposed to AuNRs were alive during measurement, which posed as a promising detection strategy in diagnosis.

### Bright and dark field microscopy of AuNRs-incubated cells

Dark-field microscopy could provide the panoramic view of the AuNRs in cells based on the light scattering from AuNRs [37]. As shown in **Figure 2F**, the AuNRs presented as bright dots in the dark-field microscopy while they appeared as black dots in the white-light microscopy. The accumulation of AuNRs in the cells presented a time dependent manner. At the early incubation stage, only several bright dots were observed in the cell. As the incubation time was elongated, amounts of the bright dots were increased correspondingly. After 3 h of incubation, the AuNRs could be clearly observed as black dots scattered in the cells under optical microscopy in white light channel, which well matched the bright dots under dark field microscope. The black dots here implied the AuNRs aggregation because single AuNRs was smaller than 100 nm and unable to be visualized with common light microscopy [1]. The aggregations were also evidenced by the black color of corresponding cell pellets after 3 hour incubation (Figure 2A) because color variation was the simplest way to distinguish AuNRs flocculation. As mentioned above, the LSPR band exhibited a gradual red shift and unsymmetrical broadening to longer wavelength region with incubation time increased (Figure 2C), which was indicative of AuNRs aggregation in the cells. The aggregation of AuNRs observed by microscopy was consistent with the variation of the spectra. Most importantly, the amounts and brightness variation of AuNRs in the dark-field microscopy exhibited the same tendency to the results from ICP-MS and TSPR peak area.

### Concentration effects of AuNRs on cells internalization

**Figure 3:**
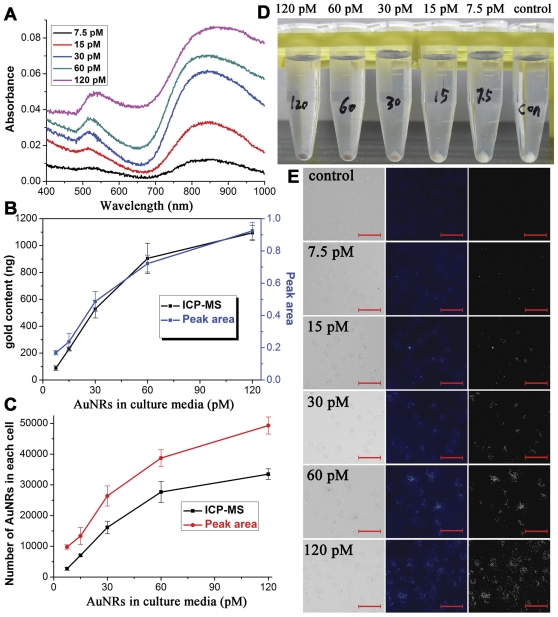
Analysis of AuNRs internalized by MDA-MB-231 cells vs AuNRs concentration. (A) Typical absorption curves of AuNRs in cells cultured with different AuNRs dosages for 6 hours. The curves are offset for clarity. (B–C) A comparison between the quantification methods of ICP-MS and TSPR peak area calculation. (D) Digital photographs of AuNRs-contained cell pellets co-cultured with different amount of AuNRs in media for 6 hours. (E) Dark field microscopic observations of cells incubated with different concentrations of AuNRs in the media. The red scale bar is 50 µm.

**Figure 4:**
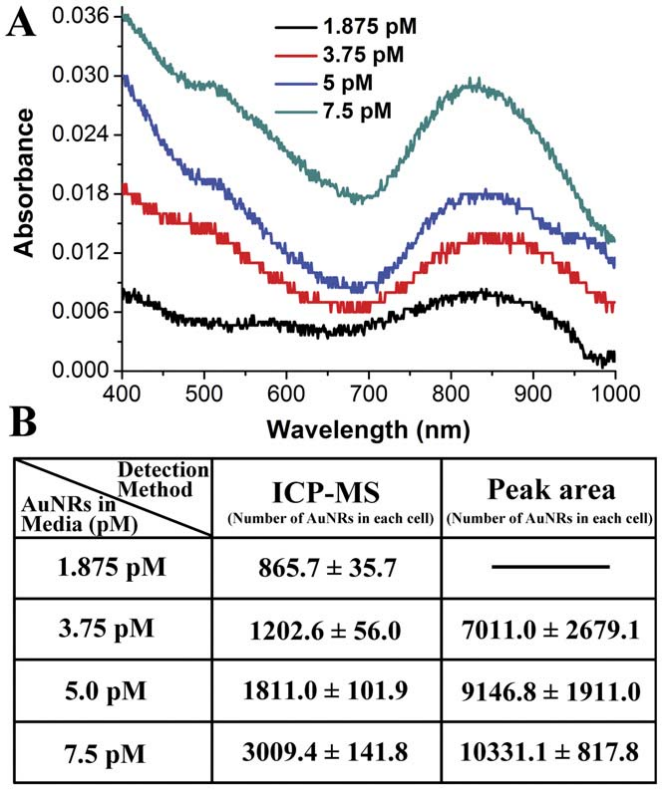
Determination of the minimum detectability of the spectroscopic analysis. (A) Representative absorption spectra of AuNRs in cells after 6-hours incubation with 1.875, 3.75, 5 and 7.5 pM AuNRs in culture media. The curves are offset for clarity. (B) Comparison between the minimum detectability of ICP-MS and TSPR peak area calculation in quantifying AuNRs in cells. The Au content in cells is converted to AuNRs number in each cell. The numbers are presented as Mean±SD from three parallel experiments.

In addition to incubation time, concentration effects of AuNRs in culture media on internalization by MDA-MB-231 cells were also directly evaluated by UV-vis-NIR absorption spectroscopy and the feasibility of this spectroscopy-based method was double-checked in reference to the results obtained from ICP-MS. In **Figure 3B**, the uptake of AuNRs obtained from UV-vis-NIR spectra and ICP-MS was shown proportionally correlated to the AuNRs concentration within 7.5–120 pM after an incubation of 6 h. This was comprehensible as a higher concentration of AuNRs around the cells surface would initiate the uptake more easily and frequently. Most importantly, the variation tendency of both content and particle number of AuNRs in the cells calculated from TSPR peak area could match those from ICP-MS analysis (**Figure 3B–C**), which strongly supported the feasibility and repeatability of this spectroscopy-based quantification for AuNRs within living cells. Besides, results from dark-field microscopy also demonstrated the quantity variation of AuNRs in the cells (**Figure 3D–E**). Furthermore, to compare the detection capability between ICP-MS and this spectroscopic analysis, the concentration of AuNRs in the culture media was reduced to 1.875 pM. After 6-hours incubation at this concentration, the AuNRs in each cell was estimated to be 865.7 nanorods detected by ICP-MS method (Figure 4). It could be seen that the TSPR peak of AuNRs in cells was too weak to be distinguished (Figure 4A), which made the peak area calculation infeasible. This was presumably resulted from the detection limitations of the absorption spectrophotometer and the absorption disturbance from the cells themselves as the absorbance of cells was relatively higher in UV-visible region than that in near-infrared region. When the AuNRs concentration was increased to 3.75 pM, the TSPR area became perceptible and the calculated AuNRs number in each cell was in proportion to that determined by ICP-MS (Figure 4B). Therefore, we would suggest the minimum detectabiliy of the spectroscopic method was 3.75 pM AuNRs in culture media for a 6-hours culture. And the corresponding minimum number of gold nanorods in each cell was about 7000 using this spectroscopic method under our experimental conditions (Figure 4B). Although the minimum concentration was higher than that of ICP-MS, its low cost and facile manipulation made it complementary to the existing characterization methods. Additionally 1.5×10^5^ of cells was needed in the spectroscopy analysis, which was regularly involved in the biochemical analysis such as western blot.

### Distribution and location of AuNRs in cells vs incubation time

**Figure 5:**
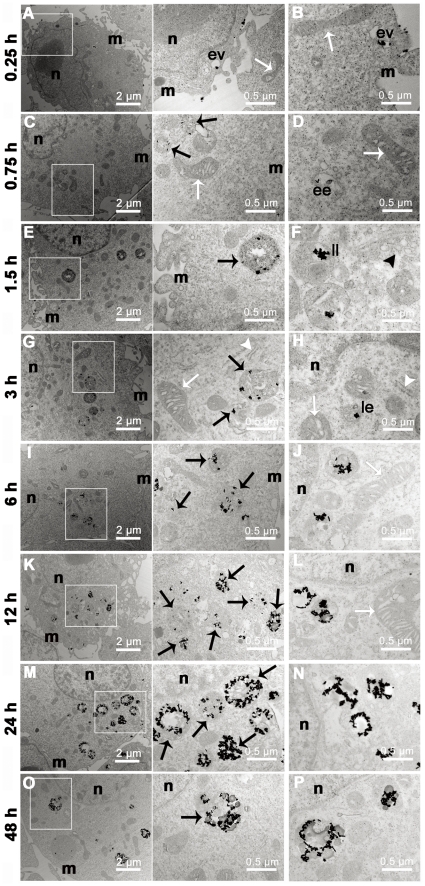
Distribution of AuNRs in MDA-MB-231 cells evaluated by TEM observation with detailed time-resolution. (A, C, E, G, I, K, M, O) The representative images of AuNRs in cells with different incubation time. The area rectangle labeled is further magnified and shown in the middle column. (B) AuNRs are shown adsorbed on the membrane and retained in the endocytic vesicle (ev). (D) AuNRs are observed in an early endosome (ee). (F) AuNRs are located in a late lysosome (ll) which is featured with multilayer structure and near Golgi apparatus. (H) AuNRs are in a late endosome (le) appeared as multivesicular body (MVB) in perinuclear region. (J, I, N, P) AuNRs-contained lysosomes located in perinuclear region after 6, 12, 24 and 48 h of incubation with AuNRs. The black arrows point lysosome, white arrows point mitochondria, and black and white arrow heads mark the Golgi apparatus and rough endoplasmic reticulum (rER), respectively. (n: nucleus, m: membrane).

In order to probe the detailed distribution of AuNRs in the cells, time resolved TEM observation was conducted and the results are presented in **Figure 5**. After an incubation of 15 minutes, AuNRs were adsorbed on the cells membrane and included in the vesicles (**Figure 5A–B**). Meanwhile AuNRs were observed to be surrounded by endocytic pits and retained in endocytic vesicles (**Figure 5B**). These observations indicated that AuNRs were internalized by MDA-MB-231 cells through endocytosis pathway because the endocytosed cargos were immediately enclosed in the vesicles [38]. After 45 and 90 minutes of incubation, AuNRs began to appear in early endosomes and lysosomes as justified from their peripheral location (**Figure 5C–F**) [39]–[40]. The lysosomatic location of AuNRs implied that endocytotic vesicles were fused with lysosomes, which was an important process involved in lysosome maturation [39]. At 3 h of incubation AuNRs trapped in late endosome were occasionally observed (**Figure 5H**), which further confirmed that AuNRs underwent an endocytic pathway that contained a series of vesicle structures including endocytic vesicle, early endosome, late endosome and lysosome, and finally resided in late lysosome [41]. With incubation time increased, most of the AuNRs were located in lysosomes while rare in vesicles. This may result from that lysosomes could fuse with endosomes in lysosome maturation [39], [42]. Given above, AuNRs were suggested to be internalized through endocytosis pathway and enter into lysosome maturation. In the maturation process, cargos in endocytic vesicles would be finally trapped in the late lysosomes which usually had a perinuclear location [41], this explained that AuNRs-contained lysosomes had a tendency of moving towards the perinuclear region (**Figure 5J–P** and **Figure S3**) which was also observed by Wei *et al* [22]. Except for endocytic vesicle system, no AuNRs were observed in other cellular compartments such as nucleus, mitochondria, Golgi apparatus, and rough endoplasmic reticulum (rER). The confinement of AuNRs in the vesicular systems may guarantee its low cytotoxicity as AuNRs could not exert a direct influence on other important organelles such as mitochondria and nucleus [19].

From whole-cell views of TEM observation, it could be seen that the uptake of AuNRs (black rods in the cells) increased with incubation time (**Figure S3**). Importantly, the AuNRs inside the cell mainly existed in the various aggregated states, in agreement with the spectral change of AuNRs in the cells. After 15 minutes incubation, AuNRs in vesicles were mainly individual or small aggregates with several AuNRs; their LSPR band was located at 794 nm, a blue shift from that dispersed in water (817 nm) (**Figure 2C**). This blue shift was considered the result of small aggregate of AuNRs as well as the change of local refractive index of AuNRs surface when they were in the cells [6], [43]. As incubation time was further increased, the AuNRs gradually accumulated in the lysosomes and formed larger aggregates during 48 h exposure. These trends were consistent with the growing red shift and broadening of LSPR band (**Figure 2C**).

### Absorption spectra of AuNRs remained in the culture media

In order to exclude the possibility that AuNRs already aggregated before entering cells, absorption spectra of AuNRs in the culture media after the incubation at each time point were also examined (**Figure S4**). No obvious broadening in the LSPR band within the incubation indicated that the AuNRs were stable and mainly existed in dispersed states in SCM [4]. The gradual aggregation of AuNRs in the cells was assumed to be a result of interactions with cell components. It has been demonstrated that pH value was gradually decreased in early endosome, late endosome and lysosome [39], which combined with harsh environment within endosomes and lysosomes may induce AuNRs flocculation [18], [44]. Besides, fusion between lysosome and endosome increased the quantity of AuNRs in a single lysosome, which would enhance their aggregation chance.

**Figure 6:**
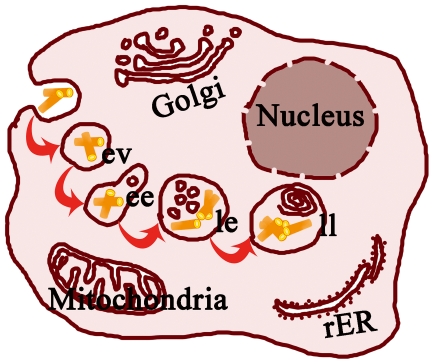
Schematic illustration of the itinerary of AuNRs in MDA-MB-231 cells. AuNRs are first taken up by endocytosis and immediately retained in endocytic vesicles (ev). The AuNRs then experience lysosome maturation process including early endosome (ee), late endosome (le) and late lysosome (ll) location. In the cell, individual AuNRs gradually aggregated one another and located towards perinuclear region. The rER and Golgi represents rough endoplasmic reticulum and Golgi apparatus, respectively.

In summary, AuNRs-contained living cells are demonstrated to harvest the absorption information of the AuNRs, thus providing a facile way to probe the behaviors of AuNRs in the cells based on simple spectroscopic method. As demonstrated in **Figure 6**, when encountered with the cells, AuNRs experienced a series of locations in the cells including endocytic vesicle, early endosome, late endosome and lysosome, and have a tendency of aggregation in the lysosomes as well as move toward perinuclear region during 48 h incubation. The uptake of AuNRs by MDA-MB-231 cells is positively correlated to both incubation time and AuNRs concentration. These findings reveal dynamic behaviors of AuNRs in cells and suggest a simple spectroscopic technique in quantifying AuNRs and characterizing their aggregation state within living mammalian cells. The variation of LSPR location in the living cells may also be helpful in optimizing laser wavelength in anti-cancer thermotherapy.

## Materials and Methods

### Materials

Silver nitrate (AgNO_3_), cetyltrimethylammonium bromide (CTAB), hydrogen tetrachloroaurate (III) trihydrate (HAuCl_4_·3H_2_O), L-ascorbic acid, glutaraldehyde (25% aqueous solution), and sodium borohydride (NaBH_4_) were obtained from Alfa Aesar. Poly (sodium-p-styrenesulfate) (PSS, molecular weight: 70000) and poly (diallyldimethyl ammoniumchloride) (PDDAC, 20%) were purchased from Sigma-Aldrich.

### Preparation of AuNRs

The as-synthesized AuNRs were coated with a bilayer of CTAB molecules. After two-step layer-by-layer electrostatic assembly, PDDAC-capped AuNRs were obtained. The AuNRs were centrifuged at 15000 rpm for 5 minutes twice and re-dispersed in deionized water as final stock solution [28]. The concentration of the AuNRs stock solution was 12 nM determined by ICP-MS and TEM measurements [45].

### Cells culture

Human breast cancer cell line MDA-MB-231 was purchased from the Cell Resource Center of Chinese Academy of Medical Sciences (Beijing, China). This cell line was well documented in 1980s and has been widely used in the breast cancer research [46]–[48]. Cells were cultured in Leibovitz's L-15 Medium (Gibco Invitrogen, CA, USA) containing phenol red, 10% fetal bovine serum (FBS), 100 U/ml penicillin, 100 U/ml streptomycin and maintained in a 37°C humidified incubator with a low-CO_2_ environment.

### Spectroscopy

Absorption spectra were recorded using BioTek Synergy™ 4 Hybrid Multi-Mode Microplate Reader (BioTek Instruments, USA); spectra were typically measured from 400 nm to 999 nm with a stepwise of 1 nm. First, fetal bovine serum was added into the Leibovitz's L-15 culture medium at different concentrations of 0%, 2.5%, 5%, 10%, 20% and 30% FBS. Bovine serum albumin (BSA) was added into the culture medium at different contents of 0.005, 0.05, 0.5, 5 and 50 g/L. The total protein amount in 5 g/L BSA in medium was estimated equal to that in 10% FBS in medium [49]. A proper amount of AuNRs was added into the prepared culture media containing different contents of FBS or BSA. The total medium volume and AuNRs concentration was fixed at 200 µl and 120 pM respectively. The corresponding medium without AuNRs was set as background. The ratio of protein to AuNRs was expressed as the number of total serum protein (TSP) or BSA per AuNRs (TSP/AuNRs or BSA/AuNRs). After 30 minutes, 24 and 48 hours incubation, the AuNRs-contained media were subjected to the UV-vis-NIR absorption spectroscopic measurements and every treatment was repeated for three times.

To acquire absorption spectra of AuNRs in the living cells, 3×10^5^ cells were seeded on to a 60-mm petri dish and incubated overnight to allow cells attachment. AuNRs were added into culture media (10% FBS) at a concentration of 60 pM. After exposed to AuNRs for different hours, the cultured medium was collected for UV-vis-NIR spectroscopy. The cells were washed by PBS buffer for 3 times to wash off the loosely bound AuNRs. Then cells were collected by trypsin digestion and following centrifugation. The cells pellets (in PBS) were photographed with a digital camera. Then the cells were diluted in an appropriate volume of PBS. Cell number was counted using TC10™ Automated Cell Counter (Biorad). Then 200 µl of cells suspension (1.5×10^5^ cells in PBS) was subjected to spectroscopic measurements. Correspondingly 200 µl of PBS was set as absorbance background and all the experiments were performed in triplicates. Meanwhile, 1.5×10^5^ cells in PBS were stocked in −20°C for later ICP-MS analysis. To examine concentration effect of AuNRs, the cells were cultured with different concentration of AuNRs for 6 h.

### Transmission electron microscopy of AuNRs-incubated cells

About 1.1×10^6^ MDA-MB-231 cells were seeded into a 10-cm petri dish and cultured overnight to allow cells adherence. Then the culture medium was changed with fresh medium containing 60 pM AuNRs. After incubation with AuNRs for 0.25, 0.75, 1.5, 3, 6, 12, 24, 48 hours, the cells were gently rinsed with PBS solution for three times to wash off the loosely bind AuNRs. The cells cultured without any AuNRs was set as control. Then cells were scraped off and centrifuged to form cell pellet in an eppendorf tube. The cell pellet was fixed in 2.5% glutaraldehyde overnight and followed by post-fixation in 1% (w/v) osmium tetroxide in phosphate buffer. After washed with phosphate buffer, the resulting pellets were dehydrated gradually by ethyl alcohol and embedded in Epon. Ultrathin sections were cut and placed on a copper meshwork. The sections were observed on a transmission electron microscope (TEM, JEM-1010) with different magnifications.

### Dark field microscopic observation

MDA-MB-231 cells were seeded and grown on coverslips in a 24-well plate (5×10^4^ cells/well). The cells were incubated with 60 pM AuNRs in medium (10% serum) for 0.25, 0.75, 1.5, 3, 6, 12, 24, 48 hours or cultured with medium which contained 0, 7.5, 15, 30, 60, 120 pM AuNRs for 6 hours. After that, the coverslips with cells were rinsed by PBS for three times and fixed in 4% paraformaldehyde for 10 minutes. After washing for three times with PBS, the coverslips were mounted with an aqueous mounting medium (Zhongshan Goldenbridge biotechnology Co, Beijing, China). The cells were visualized under a Leica microscope equipped with dark field condenser.

### ICP-MS analysis

All ICP-MS measurements were conducted on a Thermo ICP-MS XII instrument (ThermoFisher). The instrumental detection limit for gold element was as low as 7.68 pg/ml under standard laboratory conditions. The sample preparation for the ICP-MS analysis was as follows. Briefly, 1.5×10^5^ AuNRs-contained or control cells were digested by aqua regia and the final sample solutions were diluted to a fixed volume (10 ml). Each treatment was repeated for 3 times. The mass of gold element determined from ICP-MS was converted to the number of AuNRs per cell using the calculated number of gold atoms in each nanorod.

### Conversion of TSPR peak area to AuNRs number in each cell

The TSPR peak area was converted to AuNRs number in each cell through a calibration line. In order to simulate the situation of AuNRs dispersed inside the cells, AuNRs were dispersed in cell lysate of MDA-MB-231. To prepare the cell lysate, the cells was lysed by three consecutive freeze-thaw cycles followed by ultrasonication. Certain amount of AuNRs were dispersed in the cell lysate of 1.5×10^5^ cells and the total volume was fixed at 200 µl to obtain solutions with determined AuNRs number for the spectroscopic measurements. Cell lysate without AuNRs was set as control for background subtraction. The TSPR peak area of the AuNRs dispersing in the lysate was calculated according the method described in Figure S1. The calibration line of TSPR peak area vs numbers of AuNRs was created (Figure S2). The AuNRs number in each cell was calculated using the calibration line.

## Supporting Information

Figure S1 **Illustration of the TSPR peak area calculation.** The absorption spectra of AuNRs in cells were acquired by deducting the absorption from the control cells. The curves were first smoothed with the FFT (fast Fourier transform) and then a line segment connecting the inflection points before and after the TSPR peak was created. The area between the TSPR peak and the line segment was calculated in the origin 7.5 software. The inserted graph shows representative absorption spectra of AuNRs-contained and control cells.
(TIF)

Figure S2 **Calibration curve of AuNRs dispersed in MDA-MB-231 cell lysate as a function of concentration.** Before the spectroscopic analysis was performed, cells were lysed by three consecutive freeze-thaw cycles followed by ultrasonication. Various amounts of AuNRs were dispersed in cell lysate of 1.5×10^5^ cells and repeated for three times at each concentration. The inserted graph presents the representative absorption spectra of AuNRs dispersed in cell lysates.
(TIF)

Figure S3 **Whole-cell view of TEM images of MDA-MB-231cells incubated with AuNRs for various time periods.** The scale bar represents 2 µm. (A-control, B-0.25 h, C-0.75 h, D-1.5 h, E-3 h, F-6 h, G-12 h, H-24 h, I-48 h).
(TIF)

Figure S4 **Absorption of AuNRs in the culture media when cells are harvested.** (A) The UV-vis-NIR spectra of AuNRs in the media after a different incubation time. (B) The absorption spectra of AuNRs remained in the media after cultured with different concentration of AuNRs for 6 hours. The absorption disturbance around 550 nm wavelength is resulted from the strong absorption of phenol red in media when performing the background deduction [27].
(TIF)
