# Supplementary figures and images for: Probing the Behaviors of Gold Nanorods in Metastatic Breast Cancer Cells Based on UV-vis-NIR Absorption Spectroscopy
Source: PLoS One. 2012 Feb 22;7(2):e31957. doi: 10.1371/journal.pone.0031957 (PMC3284533; doi:10.1371/journal.pone.0031957)

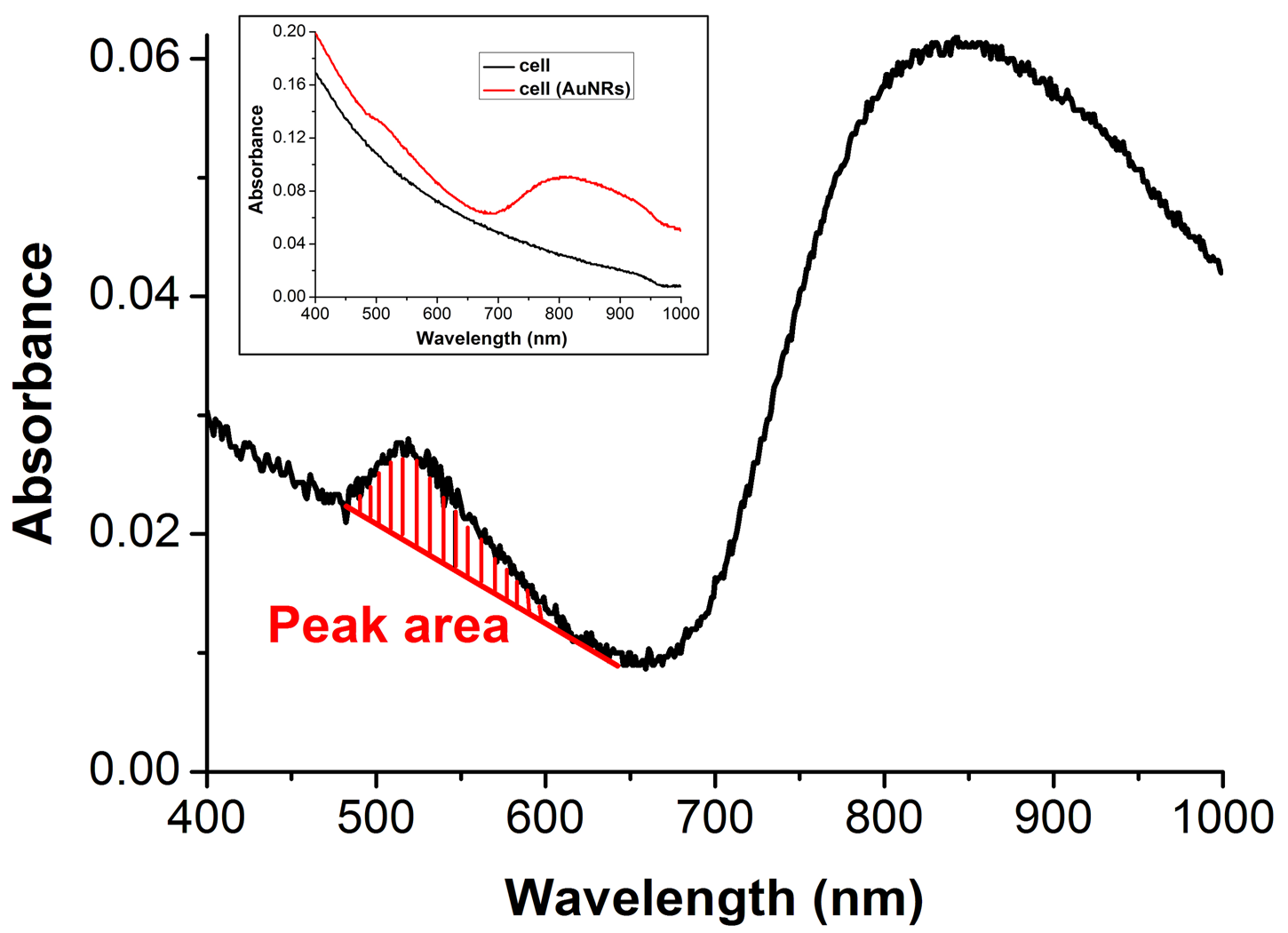

Supplement: Figure S1 — Illustration of the TSPR peak area calculation. The absorption spectra of AuNRs in cells were acquired by deducting the absorption from the control cells. The curves were first smoothed with the FFT (fast Fourier transform) and then a line segment connecting the inflection points before and after the TSPR peak was created. The area between the TSPR peak and the line segment was calculated in the origin 7.5 software. The inserted graph shows representative absorption spectra of AuNRs-contained and control cells. (TIF) [file pone.0031957.s001.tif]

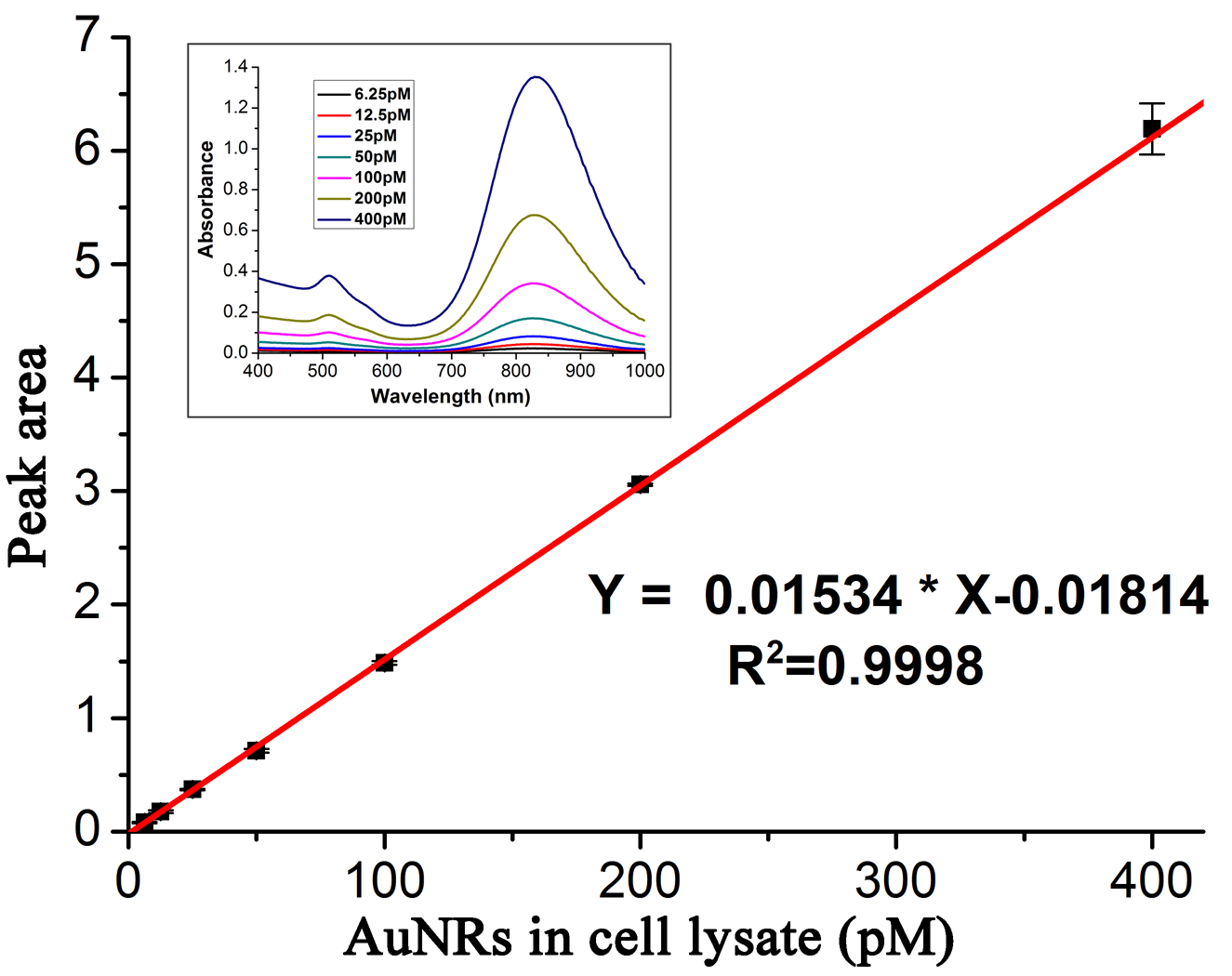

Supplement: Figure S2 — Calibration curve of AuNRs dispersed in MDA-MB-231 cell lysate as a function of concentration. Before the spectroscopic analysis was performed, cells were lysed by three consecutive freeze-thaw cycles followed by ultrasonication. Various amounts of AuNRs were dispersed in cell lysate of 1.5×10^5^ cells and repeated for three times at each concentration. The inserted graph presents the representative absorption spectra of AuNRs dispersed in cell lysates. (TIF) [file pone.0031957.s002.tif]

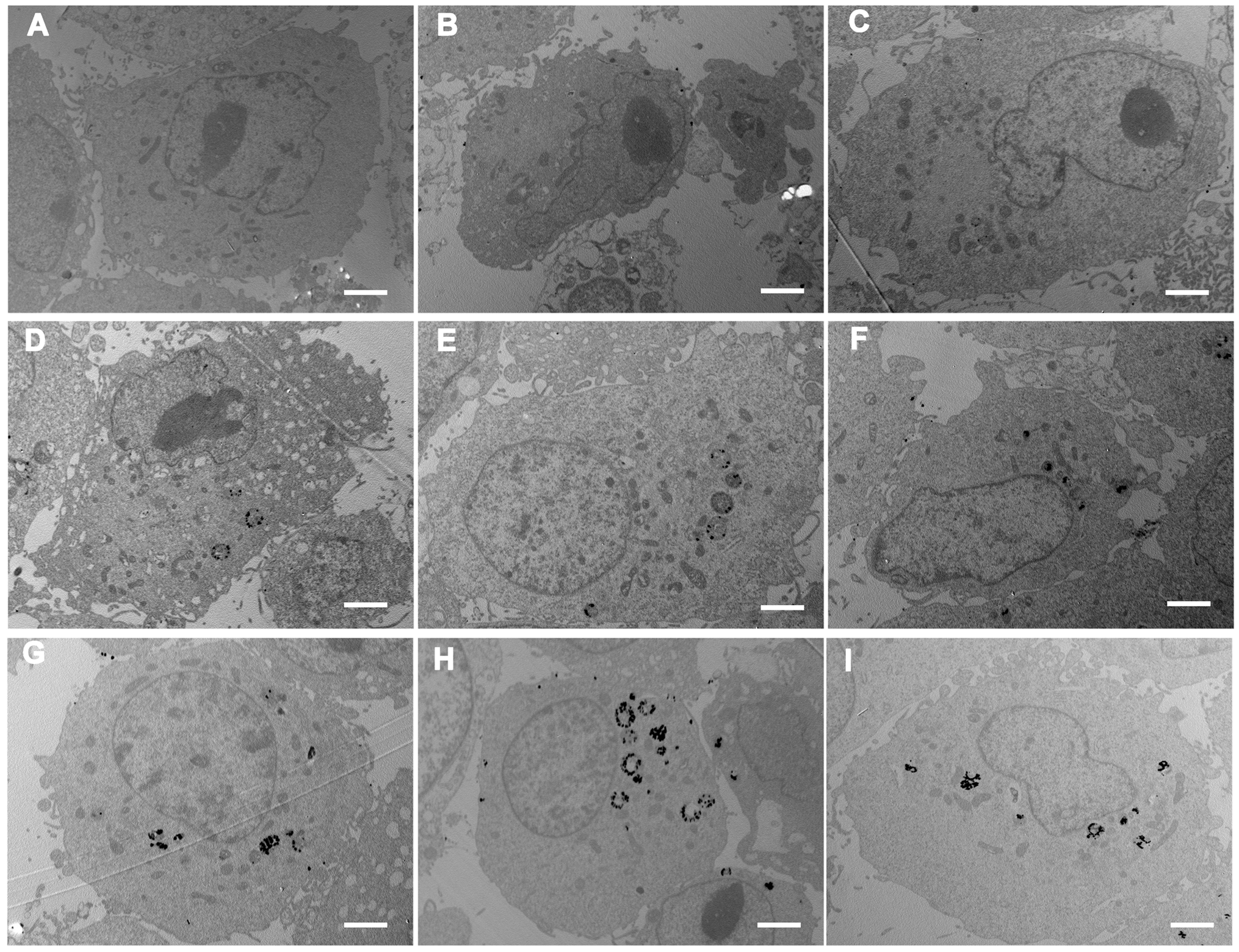

Supplement: Figure S3 — Whole-cell view of TEM images of MDA-MB-231cells incubated with AuNRs for various time periods. The scale bar represents 2 µm. (A-control, B-0.25 h, C-0.75 h, D-1.5 h, E-3 h, F-6 h, G-12 h, H-24 h, I-48 h). (TIF) [file pone.0031957.s003.tif]

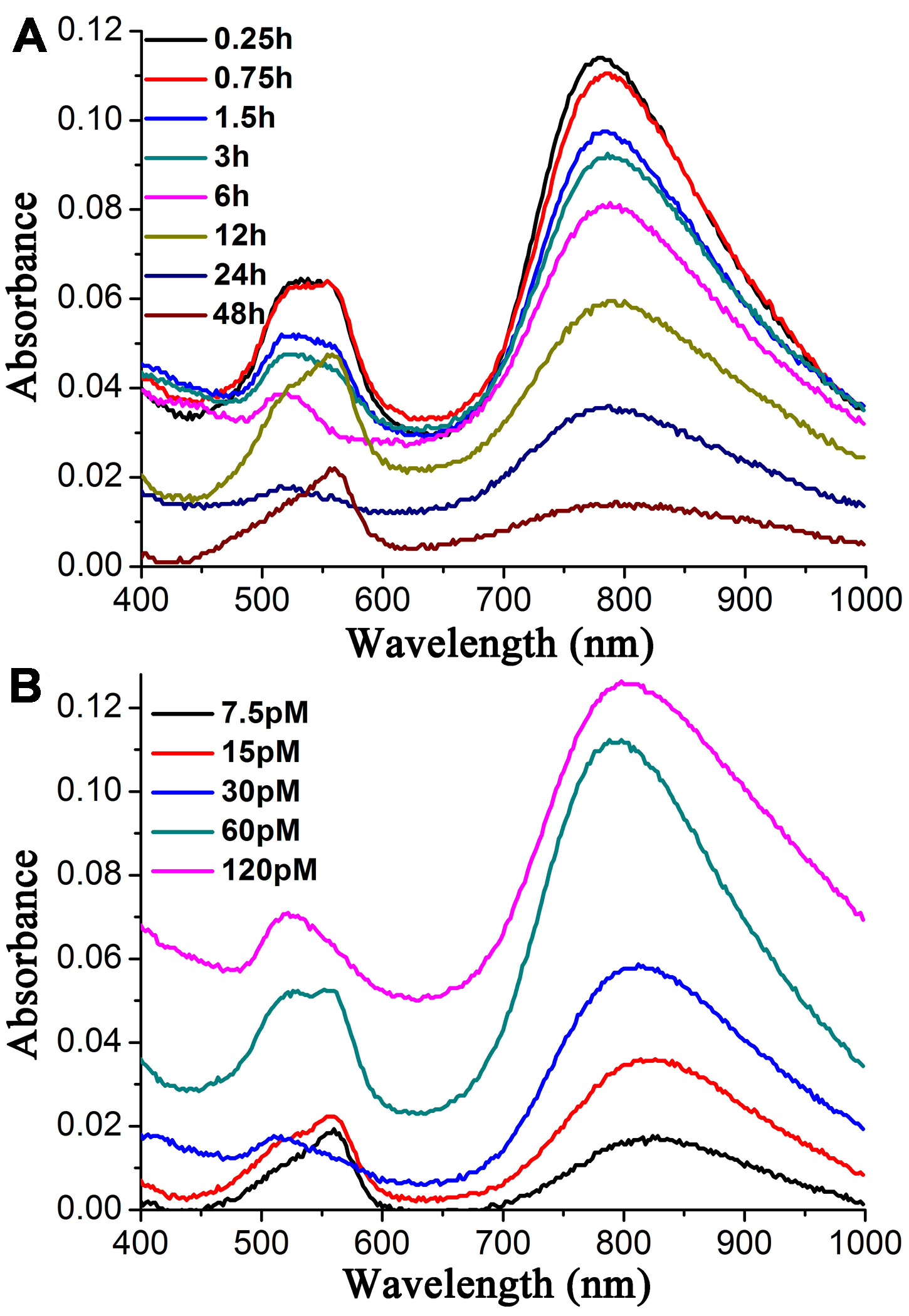

Supplement: Figure S4 — Absorption of AuNRs in the culture media when cells are harvested. (A) The UV-vis-NIR spectra of AuNRs in the media after a different incubation time. (B) The absorption spectra of AuNRs remained in the media after cultured with different concentration of AuNRs for 6 hours. The absorption disturbance around 550 nm wavelength is resulted from the strong absorption of phenol red in media when performing the background deduction [27]. (TIF) [file pone.0031957.s004.tif]
